# Cytomegalovirus mismatch after heart transplantation: Impact of antiviral prophylaxis and intravenous hyperimmune globulin

**DOI:** 10.1002/iid3.508

**Published:** 2021-09-15

**Authors:** Moritz B. Immohr, Payam Akhyari, Charlotte Böttger, Arash Mehdiani, Hannan Dalyanoglu, Ralf Westenfeld, Daniel Oehler, Igor Tudorache, Hug Aubin, Artur Lichtenberg, Udo Boeken

**Affiliations:** ^1^ Department of Cardiac Surgery Heinrich Heine University Düsseldorf Düsseldorf Germany; ^2^ Department of Cardiology Heinrich Heine University Düsseldorf Düsseldorf Germany

**Keywords:** CMV‐DNAemia, cytomegalovirus, ganciclovir, heart transplantation, intravenous hyperimmune globulin, valganciclovir

## Abstract

**Objective:**

Cytomegalovirus (CMV) infections are correlated with complications following heart transplantation (HTx) and impaired outcome. The impact of a serologic mismatch between donor and recipient and the necessity of prophylactic virostatic medication is still a matter of concern.

**Methods:**

We retrospectively reviewed all patients that underwent HTx between 2010 and 2020 in our department. The recipients (*n* = 176) could be categorized into four risk groups depending on their serologic CMV matching (D^+^/R^**−**^ = donor CMV‐IgG positive and recipient CMV‐IgG negative, *n* = 32; D^**−**^/R^+^, *n* = 51; D^**−**^/R^**−**^, *n* = 35; D^+^/R^+^, *n* = 58). All patients followed the same protocol of CMV prophylaxis with application of ganciclovir/valganciclovir and intravenous CMV hyperimmune globulin.

**RESULTS:**

Incidence of postoperative morbidity such as primary graft dysfunction, neurological events, infections, and graft rejection were comparable between all groups (*p* > .05). However, the incidence of postoperative acute kidney injury with hemodialysis was by trend increased in the D^−^/R^+^ group (72.0%) compared to the other groups. In‐hospital CMV‐DNAemia was observed in serologic positive recipients only (D^+^/R^−^: 0.0%, D^−^/R^+^: 25.0%, D^−^/R^−^: 0.0%, D^+^/R^+^: 13.3%, *p* < .01). During the first year, a total of 18 patients developed CMV‐DNAemia (D^+^/R^−^: 31.6%, D^−^/R^+^: 31.9%, D^−^/R^−^: 3.4%, D^+^/R^+^: 11.1%, *p* = .03).

**Conclusions:**

Seropositive recipients carry an important risk for CMV‐DNAemia. However, we did not observe differences in perioperative morbidity and mortality regarding CMV matching, which might be related to regularly administer prophylactic virostatics and additional CMV‐IVIG for risk constellations. For high‐risk constellation, long‐term application of CMV‐IVIG during the first year after transplant may be beneficial.

## Graphical abstract

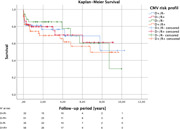

## 1 INTRODUCTION

Cytomegalovirus (CMV) infection remains a serious and common complication after orthotopic heart transplantation (HTx).^1^, ^2^ Hereby, the term CMV infection is defined as any kind of CMV replication in the body regardless of clinical symptoms and is often referred as CMV‐DNAemia in the event of a positive CMV‐PCR test.^3^ In contrast to that, CMV disease represents symptomatic CMV infection, which can be further divided into either viral syndrome with nonspecific symptoms like fever, malaise, or leucopenia or tissue invasive with end‐organ disease.^1^, ^3^

Postoperative CMV infection is associated with impaired coronary endothelial function of the graft organ and can lead to cardiac allograft vasculopathy (CAV).^4^ As a consequence, CMV infection can decrease the postoperative survival and increase the risk of organ rejection as well as infective complications.^1^, ^5^, ^6^ The risk of postoperative CMV infection is mostly correlated to the pretransplant CMV serology of the donor and recipient.^1^, ^7^, ^8^ Especially transplantation with CMV immunoglobulin G (IgG) positive donors and CMV IgG negative recipients carry a high risk of postoperative CMV infections.^1^, ^6^, ^7^, ^8^, ^9^ Therefore, postoperative prophylaxis of CMV infections by application of antiviral medication such as valganciclovir and ganciclovir is regularly used today.^1^, ^6^ In addition, application of intravenous CMV immune globulin (CMV‐IVIG) offers another potential treatment option but remains controversial because of its relatively high costs.^1^, ^6^, ^10^, ^11^ Nevertheless, postoperative CMV infections, as well as consecutive graft failure, are unfortunately still commonly observed today.^2^

Impact of serologic CMV mismatch and the optimal prophylactic antiviral treatments are still not fully understood. Therefore, we aimed to investigate the efficiency and potential beneficial impact of CMV prophylaxis with valganciclovir/ganciclovir in combination with intravenous hyperimmune globulin on the outcome after HTx.

## 2 PATIENTS AND METHODS

### 2.1 Ethics

The study followed the principles of the Declaration of Helsinki and was approved by our local university ethics committee. All patients gave their informed consent for the scientific use of anonymized patient data before inclusion in the study.

### 2.2 Patients and study design

Between 2010 and 2020, a total of *n* = 189 patients underwent HTx in our department and were prospectively enrolled in an institutional database. Patients and their corresponding donors were reviewed and retrospectively assigned to either one of four groups in regard to the serologic CMV risk profile of the recipient (donor CMV‐IgG positive and recipient CMV‐IgG negative: D^+^/R^−^, *n* = 32; donor CMV‐IgG negative and recipient CMV‐IgG positive: D^−^/R^+^, *n* = 51; donor CMV‐IgG negative and recipient CMV‐IgG negative: D^−^/R^−^, *n* = 35; donor CMV‐IgG positive and recipient CMV‐IgG positive: D^+^/R^+^, *n* = 58). The remaining patients (*n* = 13) were excluded due to incomplete data sets.

### 2.3 Study objectives and follow‐up period

All relevant recipient and donor variables were reviewed and compared between the four serologic risk groups of CMV matching. Preoperative parameters, as well as perioperative morbidity such as infective complications (bloodstream infections with pathogen detection, pneumonia, sepsis, and wound infections), acute graft rejection (>1R°), hemodialysis on ICU, and neurological complications (stroke, transient ischemic attack) as well as mortality, were analyzed and incidence of in‐hospital, first‐year and late on‐set CMV‐DNAemia, as well as survival, was examined. Mean follow‐up was 992 ± 1004 days with a minimum of 12 and a maximum of 3745 days. At the end of the study period, for all surviving patients with a minimum follow‐up period of 356 days NYHA functional status was assessed.

### 2.4 Surgical procedure and perioperative management

Included patients were transplanted in orthotopic bicaval or Shumway technique. Primary immunosuppression was administered as a combination of tacrolimus, mycophenolate mofetil, and prednisolone with the same protocol for all included patients.

### 2.5 CMV prophylaxis and treatment of CMV infections

All patients were treated following the same institutional standardized CMV prophylaxis scheme. All risk groups except D^−^/R^−^ were prophylactic treated with application of intravenous 5 mg/kg bodyweight ganciclovir per day since the second postoperative day, which was changed to oral 900 mg oral valganciclovir per day as soon as possible after extubation and continued for a total of 90 days. In case of impaired kidney function, dose was adapted following the recommended scheme by the manufacturer's package insert. In addition, patients of the high‐risk profile D^+^/R^−^ as well as patients with glomerular filtration rate <25 ml/min received an additional treatment with 50 ml (bodyweight < 75 kg) or 100 ml (bodyweight ≥ 75 kg) CMV‐IVIG (Cytotect CP^©^; Biotest AG) per day for the first 3 consecutive postoperative days. Furthermore, patients with leucopenia (white blood cells < 2.5 × 10^9^/L) and patients that were treated for acute organ rejection also regularly underwent application of 50–100 ml CMV‐IVIG application per day for 3 consecutive days. Screening for CMV‐DNAemia by PCR of blood samples was performed twice a week during the initial HTx hospital stay and pursued once a week for the first 3 postoperative months. Afterwards, PCR was done once every other week until the sixth postoperative month and once a month till the end of year one. Thenceforward, PCR was performed only once every 3 months.

**Figure 1:**
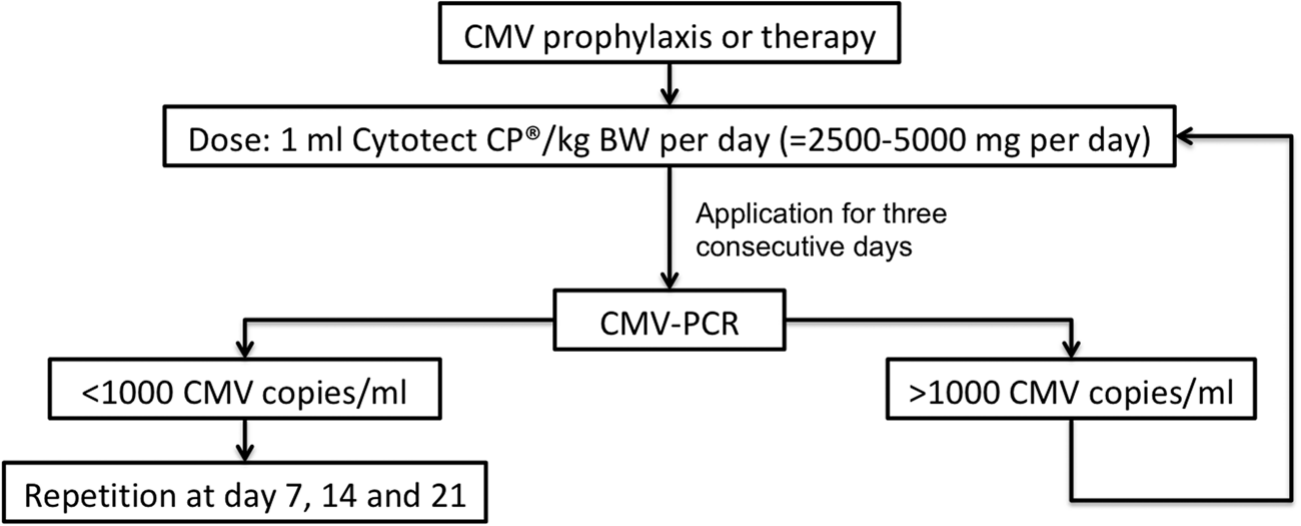
Algorithm of cytomegalovirus (CMV) prophylaxis and therapy with intravenous CMV hyperimmune globulin (Cytotect CP; Biotest AG). PCR, polymerase chain reaction

In the case of CMV‐DNAemia patients were treated with 1800 mg valganciclovir per os per day (<1000 CMV copies/ml). Patients with >1000 CMV copies/ml as well as all symptomatic cases and patients with glomerular filtration rate <25 ml/min were treated with intravenous 5 mg/kg BW ganciclovir twice a day plus 50–100 ml CMV‐IVIG per day for 3 consecutive days for in the event of continuity of CMV‐DNAemia >1000 copies/ml treatment with CMV‐IVIG was repeated once a week (Figure 1).

### 2.6 Statistics

Statistics were calculated using SPSS Statistics 26 (IBM Corporation). Two‐tailed Fisher‐Freeman‐Halton tests were used for dichotomous variables and Kruskal–Wallis tests for continuous ones. Survival analysis was performed by the Kaplan–Meier method with log‐rank test. For statistically significant results (*p* < .05), additional post‐hoc analyses by Fisher's exact test respectively a Bonferroni correction were made. All results are displayed as mean with standard deviation respectively percentage of the whole.

## 3 RESULTS

### 3.1 Recipient data

Between 2010 and 2020, a total of *n* = 189 patients underwent HTx in our department. Of those, *n* = 176 patients were included in this study and divided in four groups regarding their serologic CMV matching. Altogether, preoperative *n* = 86 recipients (48.9%) were CMV‐IgG positive. Statistical analyses revealed no statistical significant differences between the four groups in regard to important parameters of size mismatch with the corresponding donors as well as age and gender of the recipients (Table 1). Furthermore, there were no differences regarding waiting list status, the incidence of ventricular assist device support, comorbidities, and laboratory values.

**Table 1:** Preoperative recipient parameters

	D^+/^R^−^	D^−^/R^+^^ ^	D^−^/R^−^	D^+^/R^+^^ ^	*p* value
Recipient variables	(*n* = 32)	(*n* = 51)	(*n* = 35)	(*n* = 58)	
Age, y	51 ± 13	56 ± 11	54 ± 10	55 ± 10	.19
Female gender, *n* (%)	8 (25.0)	12 (23.5)	7 (20.0)	18 (31.0)	.68
Height, cm	177 ± 9	174 ± 8	176 ± 7	172 ± 9	.04^a^
Weight, kg	78 ± 17	78 ± 16	79 ± 17	77 ± 14	.92
Body mass index, kg/m^2^	24.8 ± 4.8	25.8 ± 4.5	25.5 ± 5.1	25.9 ± 4.3	.62
Predicted heart mass ratio, %	−0.89 ± 13.00	−0.69 ± 18.92	1.82 ± 15.36	−1.07 ± 18.32	.76
High urgency waitlist status, *n* (%)	14 (43.8)	29 (56.9)	18 (51.4)	30 (51.7)	.72
Ventricular assist device, *n* (%)	16 (50.0)	21 (41.2)	18 (51.4)	29 (50.0)	.74
Hemodialysis, *n* (%)	1 (3.1)	3 (6.1)	3 (8.8)	2 (3.4)	.67
Diabetes mellitus, *n* (%)	4 (12.5)	9 (17.6)	7 (20.0)	14 (24.1)	.72
Laboratory values					
Hemoglobin, g/dl	12.0 ± 2.1	12.0 ± 2.7	11.5 ± 2.1	11.8 ± 2.1	.72
Creatinine, mg/dl	1.21 ± 0.53	1.57 ± 1.57	1.55 ± 1.06	1.25 ± 0.38	.16
Bilirubin, mg/dl	0.83 ± 0.79	0.81 ± 0.60	0.82 ± 0.53	1.01 ± 1.31	.90
Lactate dehydrogenase, U/L	268 ± 134	390 ± 509	337 ± 198	360 ± 301	.38

*Note*: Preoperative recipient data in regard to the serologic CMV matching with the corresponding donors (D^+^/R^−^, donor CMV‐IgG positive and recipient CMV‐IgG negative; D^−^/R^+^, donor negative and recipient positive; D^−^/R^−^, donor and recipient negative; D^+^/R^+^, donor and recipient positive).

^a^ Post‐hoc Bonferroni correction revealed no significant results.

### 3.2 Donor data

Circulating CMV‐IgG antibodies were found in ninety donors (51.4%). Similar to the reported recipients, donor parameters did not significantly differ between the four study groups either (Table 2). Differences in mean hemoglobin values turned out to be nonsignificant after post‐hoc Bonferroni correction.

**Table 2:** Preoperative donor parameters

	D^+^/R^−^	D^−^/R^+^^ ^	D^−^/R^−^	D^+^/R^+^^ ^	*p* value
Donor variables	(*n* = 32)	(*n* = 51)	(*n* = 35)	(*n* = 58)	
Age, y	43 ± 13	40 ± 13	47 ± 12	44 ± 13	.15
Female gender, *n* (%)	12 (38.5)	21 (41.2)	15 (42.9)	30 (51.7)	.56
Height, cm	176 ± 9	176 ± 9	175 ± 8	174 ± 8	.57
Weight, kg	80 ± 17	77 ± 12	79 ± 11	78 ± 14	.74
Body mass index, kg/m^2^	25.5 ± 4.4	24.8 ± 3.2	26.0 ± 3.4	25.9 ± 4.1	.25
Ejection fraction, %	63 ± 9	59 ± 10	60 ± 10	61 ± 9	.56
Diabetes mellitus, *n* (%)	3 (9.4)	4 (7.8)	2 (5.7)	3 (5.2)	.92
Laboratory values					
Hemoglobin, g/dl	10.0 ± 2.8	11.2 ± 2.7	9.9 ± 2.5	9.8 ± 2.3	.04^a^
Lactate dehydrogenase, U/L	383 ± 225	412 ± 344	563 ± 1013	469 ± 359	.80

*Note*: Preoperative donor data in regard to the serologic CMV matching with the corresponding recipients (D^+^/R^−^, donor CMV‐IgG positive and recipient CMV‐IgG negative; D^−^/R^+^, donor negative and recipient positive; D^−^/R^−^, donor and recipient negative; D^+^/R^+^, donor and recipient positive).

^a^ Post‐hoc Bonferroni correction revealed no significant results.

### 3.3 Perioperative morbidity and mortality

**Figure 2:**
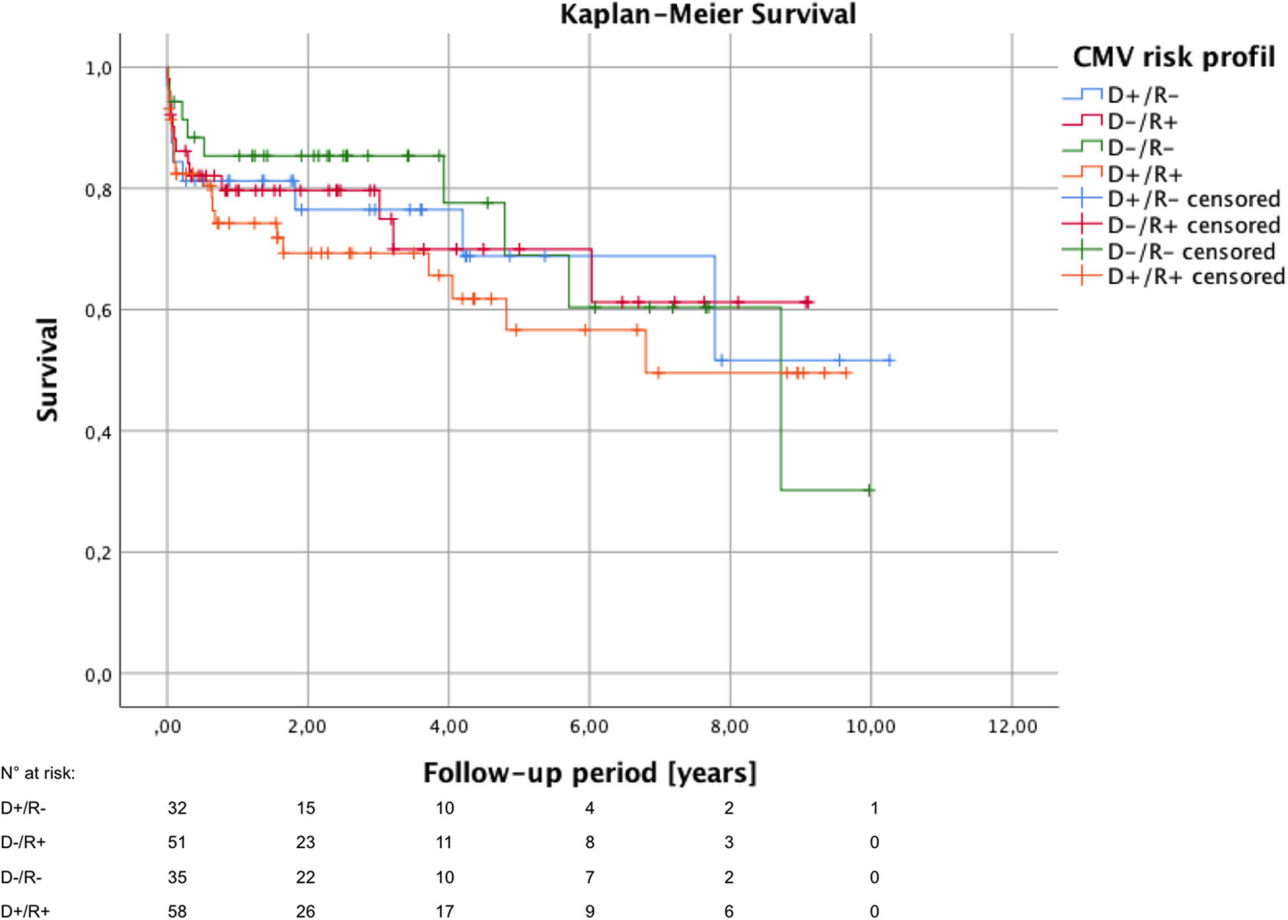
Kaplan–Meier survival analysis in regard to the serologic CMV matching (D^+^/R^−^, donor CMV‐IgG positive and recipient CMV‐IgG negative; D^−^/R^+^, donor negative and recipient positive; D^−^/R^−^, donor and recipient negative; D^+^/R^+^, donor and recipient positive. Log‐rank test: *p* = .76

Graft ischemia and postoperative hospital stay was comparable between the four risk groups (Table 3). Incidence of primary graft dysfunction with extracorporeal life support did not differ either. Common perioperative morbidity like infective complications such as pneumonia, sepsis, or wound infections, and neurological complications including stroke and transient ischemic attack as well as acute graft rejection were also equally distributed between the four groups. In contrast to that, the incidence of perioperative severe acute kidney injury with hemodialysis on ICU was by trend increased in the patient of the D^−^/R^+^ group (72.0%) compared to the others (D^+^/R^−^: 43.8%, D^−^/R^−^: 51.4%, D^+^/R^+^: 57.1%; *p* = .06). There were no significant differences in the 30‐day as well as 1‐year survival in regard to the serologic CMV matching (Table 4). These results were also confirmed by Kaplan–Meier survival analysis (*p* = .76) (Figure 2). For patients with more than 1‐year follow‐up, functional NYHA classification status was assessed at the end of the study period. The vast majority of patients were in good clinical conditions (NYHA class I) with no intergroup differences (*p* = .87).

**Table 3:** Perioperative parameters

	D^+^/R^−^	D^−^/R^+^^ ^	D^−^/R^−^	D^+^/R^+^^ ^	*p* value
Outcome variables	(*n* = 32)	(*n* = 51)	(*n* = 35)	(*n* = 58)	
Total graft ischemia time, min	234 ± 67	213 ± 51	213 ± 40	222 ± 45	.50
Transport time, min	166 ± 65	165 ± 52	147 ± 39	158 ± 43	.30
Postoperative hospital stay, d	39 ± 24	48 ± 37	44 ± 24	44 ± 37	.44
Postoperative ICU/IMC stay, d	19 ± 19	28 ± 29	23 ± 24	23 ± 28	.22
Mechanical ventilation, h	114 ± 163	202 ± 235	142 ± 170	152 ± 196	.12
Primary graft dysfunction					
Extracorporeal life support, *n* (%)	14 (43.8)	17 (33.3)	11 (31.4)	13 (22.4)	.23
Died on support, *n* (%)	4/14 (28.6)	4/17 (23.5)	1/11 (9.1)	4/12 (33.3)	.58
Blood transfusions					
Packed red blood cells, ml	3536 ± 4402	4148 ± 5299	2921 ± 3185	3830 ± 5058	.75
Platelets, ml	1625 ± 3517	1409 ± 2458	768 ± 907	951 ± 1806	.88
Fresh frozen plasma, ml	6129 ± 7676	6404 ± 6063	4780 ± 4359	6906 ± 9954	.56
Postoperative morbidity					
Infective complications, *n* (%)	5/32 (15.6)	17/49 (43.7)	6/35 (17.1)	15/55 (27.3)	.17
Acute graft rejection, *n* (%)	1/32 (3.1)	4/49 (8.2)	4/35 (11.4)	4/55 (7.3)	.69
Hemodialysis on ICU, *n* (%)	14/32 (43.8)	36/50 (72.0)	18/35 (51.4)	32/56 (57.1)	.06
Neurological complications, *n* (%)	6/32 (18.8)	11/50 (22.0)	6/35 (17.1)	9/55 (16.4)	.90

*Note*: Perioperative parameters in regard to the serologic CMV matching with the corresponding recipients (D^+^/R^−^, donor CMV‐IgG positive and recipient CMV‐IgG negative; D^−^/R^+^, donor negative and recipient positive; D^−^/R^−^, donor and recipient negative; D^+^/R^+^, donor and recipient positive).

**Table 4:** CMV status and postoperative outcome

	D^+^/R^−^	D^−^/R^+ ^	D^−^/R^−^	D^+^/R^+^^ ^	*p* value
Outcome variables	(*n* = 32)	(*n* = 51)	(*n* = 35)	(*n* = 58)	
CMV infection, *n* (%)					
In‐hospital CMV‐DNAemia, *n* (%)	0/26 (0.0)	10/40 (25.0)	0/32 (0.0)	6/45 (13.3)	<.01
1‐year CMV‐DNAemia, *n* (%)	6/19 (31.6)	7/32 (21.9)	1/29 (3.4)	4/36 (11.1)	.03
1‐year CMV disease, *n* (%)	2/6 (33.3)	0/7 (0.0)	0/1 (0.0)	0/4 (0.0)	.25
Post 1‐year CMV‐DNAemia, *n* (%)	3/19 (15.8)	4/28 (14.3)	0/28 (0.0)	6/34 (17.6)	.09
30‐day survival, *n* (%)	28/32 (87.5)	44/50 (88.0)	33/35 (94.3)	49/56 (87.5)	.74
1‐year survival, *n* (%)	21/27 (77.8)	29/39 (74.4)	28/32 (87.5)	33/46 (71.7)	.41
Longer‐term functional outcome					.87
NYHA class I, *n* (%)	14/18 (77.8)	17/25 (68.0)	16/23 (69.6)	20/28 (71.4)	
NYHA class II, *n* (%)	3/18 (16.7)	6/25 (24.0)	3/23 (13.0)	4/28 (14.3)	
NYHA class III, *n* (%)	1/18 (5.6)	2/25 (8.0)	4/23 (17.4)	4/28 (14.3)	
NYHA class IV, *n* (%)	0/18 (0.0)	0/25 (0.0)	0/23 (0.0)	0/28 (0.0)	

*Note*: Postoperative parameters in regard to the serologic CMV matching with the corresponding recipients (D^+^/R^−^, donor CMV‐IgG positive and recipient CMV‐IgG negative; D^−^/R^+^, donor negative and recipient positive; D^−^/R^−^, donor and recipient negative; D^+^/R^+^, donor and recipient positive. Longer‐term functional outcome represents New York Heart Association (NYHA) functional classification at last follow‐up during the study period for patients currently alive with more than 1‐year follow.

### 3.4 Incidence of postoperative CMV infection

Routine PCR of ethylenediaminetetraacetic acid blood samples during the initial HTx hospital stay revealed CMV‐DNAemia in preoperative seropositive recipients only (Table 4). The highest incidence (25.0%) was observed in D^−^/R^+^. After hospital discharge and till the end of the first postoperative year, CMV‐DNAemia was observed in a total of 18 of 116 patients (15.5%) with the highest incidence in the D^+^/R^−^ risk group (31.6%) and lowest in D^−^/R^−^ (3.4%). Only two patients suffered from CMV disease with both diarrheas as the only symptom. After the end of the first postoperative year, CMV‐DNAemia was observed in a total of 13 of 109 patients (11.9%) with comparable incidence in the D^+^/R^−^ (15.8%), D^−^/R^+^ (14.3%), and D^+^/R^+^ (17.6%) groups but not a single case in the D^−^/R^−^ group (*p* = .09).

## 4 DISCUSSION

CMV infections carry a relevant risk of impaired postoperative outcome after HTx by promoting several complications such as CAV, graft rejection, and infections.^1^, ^6^ In this retrospective analysis of 176 consecutive patients undergoing HTx in our department in a 10‐year study period, we reviewed the impact of our CMV prophylaxis scheme with valganciclovir/ganciclovir and CMV‐IVIG.

We tried to improve the antiviral prophylaxis by additional application of CMV‐IVIG in patients with increased risk of CMV infections, especially patient with impaired kidney function who carry an increased risk for CMV infections due to decreased doses of antiviral medications.^12^ About half of all our donors and recipients were preoperatively CMV‐IgG positive, which is comparable with the prevalence of about 60% seropositive adults in developed countries that is described in the literature.^13^ Recipients and donors of the four serologic risk profiles were comparable in regard the their demographics and common risk factors for impaired posttransplant survival, that could have influenced the results as a potential confounder.^14^

In our cohort, the CMV matching of the recipients affected neither intensive care unit nor total postoperative hospital stay. As CMV infections are a risk factor of posttransplant morbidity, they are in general associated with increased hospitalization time and costs for the health care system.^15^ The incidence of postoperative infections other than CMV infections, for example, bacterial pneumonia, sepsis, and wound infections is also increased by CMV.^1^, ^16^ In line with the literature, with our prophylaxis protocol, we were able to prevent this indirect adverse effect of posttransplant CMV infections in our cohort, indicated by comparable incidences for risk constellation with prophylaxis and D^−^/R^−^ without.^17^ CMV infection increases the risk for acute graft rejection after heart transplant even in patients with prophylactic antiviral medication.^2^ In contrast to that, we did not observed increased rates of acute graft rejection in serologic CMV risk constellations compared to the D^−^/R^−^ control group without prophylaxis.

Incidence of postoperative acute kidney failure with dialysis on intensive care unit was by trend increased in patients with D^−^/R^+^ risk profile (*p* = .06). However, the reason for this effect remains unclear. Incidence of preoperative dialysis therapy was only slightly elevated in this group compared to the others. An association with CMV‐IVIG applications could be most likely excluded as it was only administered secondary after occurrence of renal failure and was not elevated in the D^+^/R^−^ group with regularly CMV‐IVIG application. In addition there is no such correlation reported in the literature and application of CMV‐IVIG is also common prophylaxis strategy after kidney transplant.^18^

CMV mismatch did also not impact on the survival and clinical functional status after HTx in our cohort. This is contrary to the results reported for patients with CMV mismatch and missing prophylaxis as well as for patients with isolated ganciclovir prophylaxis.^8^ In addition, Stern and colleagues even reported impaired survival for patients with valganciclovir prophylaxis.^2^ In contrast to that, in line with our results, prophylactic application of CMV‐IVIG was associated with increased survival in pediatric heart transplant.^10^

Finally, incidence of in‐hospital CMV infection in high‐risk D^+^/R^−^ was significantly decreased by our prophylaxis protocol without any case of detected CMV‐DNAemia, which is obviously superior to the data reported in the literature.^7^, ^8^, ^9^ However, this effect vanished considering the longer‐term data. After completion of the regular prophylaxis 90 days after HTx, especially in patients with high‐risk profiles new CMV infections were detected. This is confirmed by the literature and questions the recommendation for only 3 months prophylaxis by the International Society of Heart and Lung Transplantation.^1^, ^9^

## 5 LIMITATIONS

The study is limited by its retrospective and single‐center design. Due to the relative long inclusion period of 10 years, follow‐up period of the patients varies a lot. As in general, the follow‐up period is relatively short, survival analysis by Kaplan–Meier method (Figure 2) most likely overestimate the mid‐ and long‐term mortality during follow‐up caused by the typical disproportionately high first‐year mortality after HTx and high number of early censored patients due to a short follow‐up period. Nonetheless, we reported a comparable cohort of the four serologic risk groups and were able to show the impact of our department's CMV prophylaxis scheme on the outcome after HTx and the incidence of postoperative CMV‐DNAemia in particular.

## 6 CONCLUSIONS

CMV infections remain a serious complication after HTx and can impair the postoperative outcome especially within the early postoperative period. Mismatch of donor and recipient CMV serology did not impact on the postoperative outcome, which might be linked to the prophylactic virostatic therapy as well as the administering of CMV hyperimmune globulin in high‐risk constellations and vulnerable patients. For high‐risk constellations, we suggest a repetition of the CMV‐IVIG treatment throughout the first year after HTx, as CMV‐DNAemia was observed more often after hospital discharge and completion of the regular prophylaxis.

## CONFLICT OF INTERESTS

The authors declare that there are no conflict of interests.

## AUTHOR CONTRIBUTIONS

*Conceptualization, data curation, formal analysis, investigation, methodology, validation, visualization, writing—original draft, writing—review* and *editing*: Moritz B. Immohr. *Conceptualization data curation, investigation, methodology, project administration, resources, supervision, validation, writing—review* and *editing*: Payam Akhyari. *Data curation, validation, writing—review* and *editing*. Arash Mehdiani. *data curation, validation, writing—review* and *editing*: Charlotte Böttger. *Data curation, validation, writing—review* and *editing*: Hannan Dalyanoglu. *Data curation, validation, writing—review* and *editing*: Ralf Westenfeld. *Data curation, validation, writing—review* and *editing*: Daniel Oehler. *Data curation, validation, writing—review* and *editing*: Igor Tudorache. *Data curation, validation, writing—review* and *editing*: Hug Aubin. *Conceptualization data curation, investigation, methodology, project administration, resources, supervision, validation, writing—review* and *editing*: Artur Lichtenberg. *Conceptualization data curation, investigation, methodology, project administration, resources, supervision, validation, writing—review and editing*: Udo Boeken.

## Data Availability

The data that support the findings of this study are available from the corresponding author upon reasonable request.
